# Transcriptional and proteomic insights into the host response in fatal COVID-19 cases

**DOI:** 10.1073/pnas.2018030117

**Published:** 2020-10-20

**Authors:** Meng Wu, Yaobing Chen, Han Xia, Changli Wang, Chin Yee Tan, Xunhui Cai, Yufeng Liu, Fenghu Ji, Peng Xiong, Ran Liu, Yuanlin Guan, Yaqi Duan, Dong Kuang, Sanpeng Xu, Hanghang Cai, Qin Xia, Dehua Yang, Ming-Wei Wang, Isaac M. Chiu, Chao Cheng, Philip P. Ahern, Liang Liu, Guoping Wang, Neeraj K. Surana, Tian Xia, Dennis L. Kasper

**Affiliations:** ^a^Institute of Pathology, Tongji Hospital, Tongji Medical College, Huazhong University of Science and Technology, 430074 Wuhan, P. R. China;; ^b^Department of Research and Development, Hugobiotech Co., Ltd., 100000 Beijing, P. R. China;; ^c^School of Automation Science and Engineering, Faculty of Electronic and Information Engineering, Xi’an Jiaotong University, 710049 Xi’an, P.R. China;; ^d^Department of Pathology, School of Basic Medicine, Tongji Medical College, Huazhong University of Science and Technology, 430074 Wuhan, P. R. China;; ^e^Department of Pediatrics, Duke University School of Medicine, Durham, NC 27710;; ^f^Department of Molecular Genetics and Microbiology, Duke University School of Medicine, Durham, NC 27710;; ^g^Institute of Artificial Intelligence, Huazhong University of Science and Technology, 430074 Wuhan, P. R. China;; ^h^Department of Pathology, Union Hospital, Tongji Medical College, Huazhong University of Science and Technology, 430030 Wuhan, P. R. China;; ^i^The National Center for Drug Screening, Shanghai Institute of Materia Medica, Chinese Academy of Sciences, 201203 Shanghai, P. R. China;; ^j^Department of Medicine, Baylor College of Medicine, Houston, TX 77030;; ^k^Department of Cardiovascular and Metabolic Sciences, Cleveland Clinic Lerner Research Institute, Cleveland, OH 44195;; ^l^Department of Forensic Medicine, Tongji Medical College, Huazhong University of Science and Technology, 430030 Wuhan, P. R. China;; ^m^Department of Immunology, Duke University School of Medicine, Durham, NC 27710;; ^n^Department of Immunology, Blavatnik Institute, Harvard Medical School, Boston, MA 02115

**Keywords:** COVID-19, SARS-CoV-2, neutrophil, fibrosis, NETosis

## Abstract

Coronavirus disease 2019 (COVID-19), the global pandemic caused by SARS-CoV-2, has resulted thus far in greater than 933,000 deaths worldwide; yet disease pathogenesis remains unclear. Clinical and immunological features of patients with COVID-19 have highlighted a potential role for changes in immune activity in regulating disease severity. However, little is known about the responses in human lung tissue, the primary site of infection. Here we show that pathways related to neutrophil activation and pulmonary fibrosis are among the major up-regulated transcriptional signatures in lung tissue obtained from patients who died of COVID-19 in Wuhan, China. Strikingly, the viral burden was low in all samples, which suggests that the patient deaths may be related to the host response rather than an active fulminant infection. Examination of the colonic transcriptome of these patients suggested that SARS-CoV-2 impacted host responses even at a site with no obvious pathogenesis. Further proteomics analysis validated our transcriptome findings and identified several key proteins, such as the SARS-CoV-2 entry-associated protease cathepsins B and L and the inflammatory response modulator S100A8/A9, that are highly expressed in fatal cases, revealing potential drug targets for COVID-19.

## Significance

Although the overwhelming majority of COVID-19-related deaths are due to respiratory failure, little is known about the host response to SARS-CoV-2 in lung parenchyma. By profiling the lung and colon transcriptome and lung proteome of nine patients who died of COVID-19 during the first wave of the pandemic in Wuhan, China, we obtained molecular insights into the host response to severe SARS-CoV-2 infection. Interestingly, all samples had a low viral burden, a finding that suggests the patients’ deaths may be due to uncontrolled host inflammatory processes rather than an active viral infection. Taken together, our findings shed light on COVID-19 pathophysiology and offer potential therapeutic targets for severe COVID-19 disease.

SARS-CoV-2, the etiologic agent for coronavirus disease 2019 (COVID-19), emerged in Wuhan, China, in late December 2019 (1). Since then, more than 29.5 million people have been infected worldwide, with over 933,000 deaths as of September 15, 2020. Although research into the clinical and scientific features of SARS-CoV-2 pathogenesis has progressed at a dizzying pace, pathogenesis of COVID-19 is still incompletely understood. Previous studies have noted profound SARS-CoV-2-induced transcriptional and immunological changes in cell culture and animal models (2, 3), as well as in human blood (4), nasopharyngeal samples (5), and bronchoalveolar lavage fluid (6). However, it is notable how little is known about the host response to SARS-CoV-2 in human lung tissue given that COVID-19 deaths are primarily due to pneumonia-related complications.

## Results

### Lung Transcriptome Response in Fatal Cases.

**Fig. 1.**
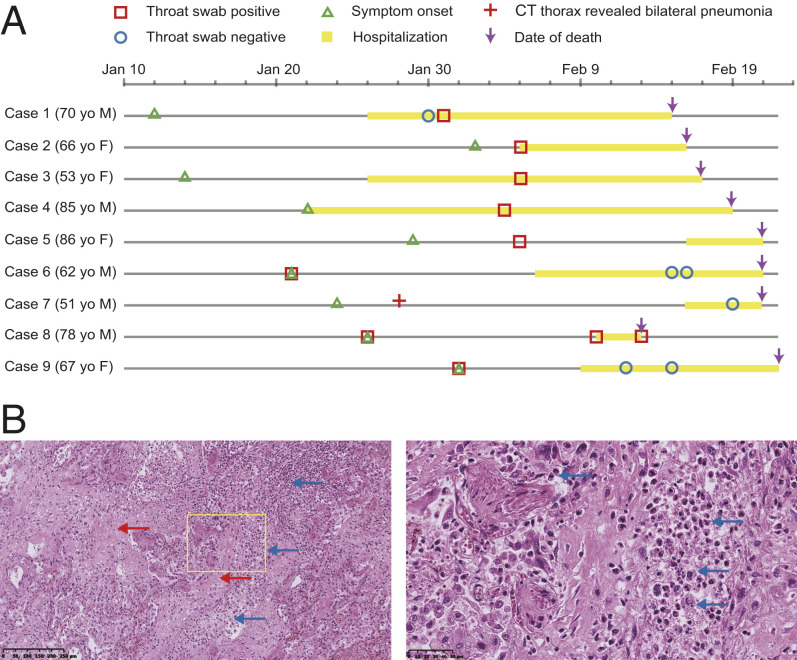
An overview of the patient cohort from Wuhan, China. (*A*) Schematic highlighting a timeline of illness onset, SARS-CoV-2 RNA detection, hospitalization, and death for the cases. Patients are aligned by the calendar dates indicated at the *Top* of the schematic. The age and sex of each patient are also listed. (*B*) Representative hematoxylin and eosin-stained lung tissue section. Areas of fibrosis (red arrows) and infiltration by neutrophils and macrophages (blue arrows) are highlighted. The image on the *Right* represents a magnified view of the area highlighted by the yellow box.

We obtained postmortem lung samples from nine patients who died of COVID-19 during the initial outbreak in Wuhan, China. The patients were symptomatic for an average of ∼26 d (range, 14 to 34 d) prior to death (Fig. 1*A*). Given that this was early in the outbreak, there was not a standard approach to treating patients, and they received a variety of antimicrobial and immunomodulatory medications as presented in Dataset S1, along with other detailed clinical findings. Consistent with other autopsy series (7–9), all patients had histological evidence of diffuse alveolar damage, with widespread hyaline membrane formation, evidence of fibrosis, and varying degrees of an inflammatory infiltrate. Notably, several patients were noted to have a significant number of neutrophils present in the lung tissue (Fig. 1*B*).

**Fig. 2.**
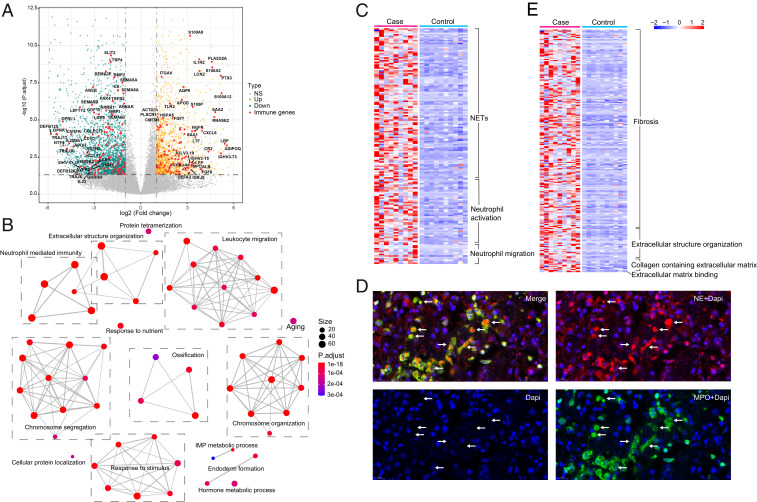
Transcriptional profiling reveals enrichment of neutrophil and lung fibrosis gene pathways. (*A*) Volcano plot (*P* value vs. fold change) comparing gene expression in cases vs. controls. Up- (orange) and down-regulated genes (cyan) are highlighted, and immune-related genes that are differentially expressed are named. The horizontal dashed line represents an adjusted *P* value of 0.05 (Wald test in DESeq2, multiple test correction by Benjamini–Hochberg [BH]), and the vertical dashed lines represent log2FC of −1 and 1. (*B*) GO network analysis of the top 60 enriched GO terms in the up-regulated genes (Fisher’s exact test using enrichGO function in R package clusterProfiler, multiple test correction by BH). (*C*) Heatmap of the up-regulated DEGs (Wald test in DESeq2, multiple test correction by BH) involved in the GO network module neutrophil-mediated immunity. (*D*) Immunofluorescence microscopy of lung section stained with DAPI (blue) and antibodies against myeloperoxidase (green) and neutrophil elastase (red). Areas of NETosis are highlighted with arrows. (*E*) Heatmap of the up-regulated DEGs (Wald test, multiple test correction by BH) involved in the GO network module extracellular structure organization.

To better understand the molecular basis underlying fatal COVID-19 cases, we performed total RNA sequencing (RNA-Seq) on the formalin-fixed paraffin-embedded (FFPE) lung samples from these 9 fatal cases; as controls, we used archived histologically normal lung tissue obtained from 10 SARS-CoV-2-uninfected individuals that underwent biopsy or surgical resection as part of routine clinical care for lung cancer. Consistent with these control samples representing noncancer-affected tissue, we did not observe enrichment of any cancer-related pathways. We identified a total of 4,065 differentially expressed genes (DEGs), with 1,470 up-regulated and 2,595 down-regulated genes in the COVID-19 patients (Fig. 2*A*). There were numerous immune-related genes affected, some of which are highlighted in Fig. 2*A*. Although patients with severe COVID-19 have been found to have a cytokine storm in peripheral blood and bronchoalveolar lavage fluid (6, 10), we found in lung parenchyma a roughly equal number of up- and down-regulated genes that are related to cytokines, chemokines, and their receptors (Dataset S2). Notably, expression of interleukin (IL)-6 was unchanged in the lungs of patients with COVID-19 (*SI Appendix*, Fig. S1), a finding that raises a question about the potential for anti-IL-6 therapy to help resolve the pulmonary inflammatory process in patients with severe disease.

Given the profound degree of transcriptional changes observed, we sought to determine how much virus was present within these lung tissue samples. We recovered viral reads from the sequencing data and mapped them to the reference viral genome of SARS-CoV-2 isolate Wuhan-Hu-1 (11). Surprisingly, even though these patients all died of COVID-19 pneumonia and displayed a significant transcriptional response relative to healthy controls, there were very few viral reads present in the samples (Dataset S3). Out of an average of 98.2 million reads per sample (Dataset S4), we recovered only 2 to 177 viral reads in six of the cases. The other three cases had no reads that mapped to the SARS-CoV-2 genome at all; however, directed SARS-CoV-2 RT-PCR of the input RNA used for RNA sequencing confirmed the presence of virus in these samples at low abundance (*SI Appendix*, Fig. S2). This finding of little virus being present in lung tissue at the time of death suggests that these patients may have died of sequelae related to COVID-19 pneumonia (i.e., the host inflammatory response) rather than an ongoing, fulminant active viral infection.

### Neutrophil Activation and Pulmonary Fibrosis Are Major Contributors to the Pathogenesis in the Lung.

To systematically identify biological pathways representative of the host tissue response in COVID-19, we performed gene ontology (GO) enrichment analysis of significantly up- and down-regulated genes. The top 60 enriched GO terms were further organized into a network with edges connecting overlapping gene sets. The functional modules were identified as the mutually overlapping gene sets clustered together and named using GO hierarchical structure terms (Fig. 2*B*). Some of the modules identified, such as “leukocyte migration” and “chromosome segregation” (the latter of which contains gene sets relevant to mitosis and cell division), are not unexpected in response to severe infection. Other modules, however, seemed less intuitive as a response to a viral pneumonia. The top two hits from the GO analysis of up-regulated genes reflected neutrophil activation and neutrophil-mediated immunity (*SI Appendix*, Fig. S3*A*), a finding that is consistent with the neutrophilic infiltrate observed upon histologic assessment of these tissues. Closer inspection of the up-regulated genes in the neutrophil activation pathways revealed many genes involved in the generation of neutrophil extracellular traps (NETs), such as myeloperoxidase, lactoferrin, and histones (Fig. 2*C* and Dataset S5). NETs are known to form in the setting of infections as a defense mechanism against pathogens, including viruses (12); however, dysregulated NETosis triggered by platelets can lead to ongoing tissue damage and thrombosis (13, 14). Patients with COVID-19 have been found to have increased serum levels of myeloperoxidase–DNA complexes and citrullinated histone H3, both of which are markers of NETs (15). Using immunofluorescence, we identified foci where myeloperoxidase, neutrophil elastase, and cytoplasmic DNA were colocalized (Fig. 2*D*), an observation that demonstrates the presence of NETs in the lungs of fatal COVID cases. Given the paucity of viral RNA present in these samples and the overwhelming degree of pulmonary thromboses identified during the autopsies of these patients, it is possible that these NETs have been triggered by platelets rather than SARS-CoV-2. Consistent with this hypothesis, expression of platelet factor 4, which is often involved in platelet-triggered NETosis (16), is increased within the cases by ∼5.5-fold (*SI Appendix*, Fig. S4).

Another module identified in our network analysis that seemed less clearly relevant to a severe viral infection was “extracellular structure organization.” We reasoned, however, that this gene cluster might reflect the hyaline membrane formation and fibrosis seen on histology. When we interrogated the function of these genes further, we found that many of them (e.g., MMP7, GDF15, and MUC5B) have been implicated in the pathogenesis of pulmonary fibrosis (17) (Fig. 2*E* and Dataset S6). Indeed, many of these patients had been symptomatic for nearly a month prior to their ultimate demise, and they may have begun to enter the fibrotic phase of diffuse alveolar damage (18). Moreover, it has been noted that patients with severe COVID-19 develop a more typical picture of acute respiratory distress syndrome with eventual loss of lung compliance (19). Considered together, our findings help bridge the clinical and histological observations with their molecular bases.

Surprisingly, the top three GO terms that were down-regulated in the lung represent neurobiological processes: axon guidance, neuron projection guidance, and synapse organization (*SI Appendix*, Fig. S3*B*). Expression of several semaphorins and their plexin receptors, genes that play a critical role in axon guidance, as well as airway development and disease (20), were down-regulated, as were Ephrins and their Eph receptors, which are expressed in lung epithelial cells and help regulate T cell activation (21, 22) (*SI Appendix*, Fig. S5*A*). Additionally, we observed down-regulated expression of three genes that are markers for pulmonary neuroendocrine cells (PNECs), which are specialized epithelial cells important in airway sensory function that are innervated by neurons (*SI Appendix*, Fig. S5*B*). Intriguingly, defects in PNECs can lead to altered cytokine production and increased lung inflammation (23, 24). Broadly, there is growing evidence of neuroimmune interactions in the lung (25, 26), and the down-regulation of these pathways may reflect an immunomodulatory process. Future work will be needed to determine whether distinct lung-innervating neuronal subtypes or their synaptic nerve terminals are affected in lung tissues following infection.

### Colon Transcriptome Response in Fatal Cases.

**Fig. 3.**
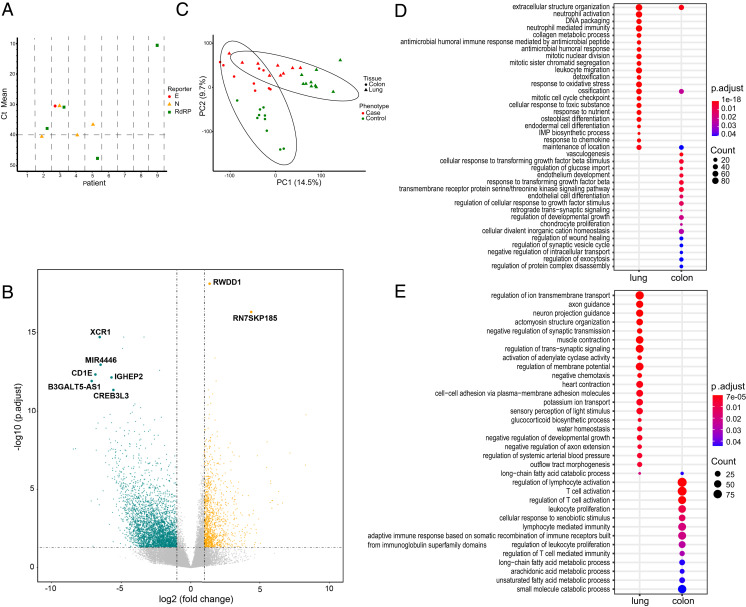
Transcriptional profiling reveals altered gene expression in the colon of COVID-19 deceased patients. (*A*) SARS-CoV-2 RT-PCR results from the RNA input for transcriptional profiling. The three genes assayed include the nucleocapsid (N), envelope (E), and RNA-dependent RNA polymerase (RdRp) genes. The dotted line indicates the threshold for detecting a gene as present. (*B*) Volcano plot (*P* value vs. fold change) comparing gene expression in cases vs. controls. Up- (orange) and down-regulated genes (cyan) are highlighted, and genes differentially expressed with |logFC| > 5 and −log10 padj > 10 (down-regulated genes) or >15 (up-regulated genes) are named. (Wald test in DESeq2, multiple test correction by BH). (*C*) Principal component analysis of normalized read counts for the entire transcriptome. Ellipses indicate 95% confidence interval of group membership. Percentages along the axes indicate the degree of variance explained by that principal component. (*D* and *E*) Dotplot visualization of the top 20 enriched GO terms of up-regulated (*D*) and down-regulated (*E*) DEGs in the lung and colon of COVID-19 deceased patients. The color of the dots represents the *P* value adjusted by Benjamini–Hochberg correction for each enriched GO term identified by Fisher’s exact test using enrichGO function in R package clusterProfiler, and the size of the dot represents the number of genes enriched in the total gene set.

A total of 2 to 15% of patients with COVID-19 had gastrointestinal symptoms such as diarrhea, abdominal pain, and vomiting (27). None of the nine patients had any gastrointestinal symptoms based on their medical records; however, we did detect all of the three marker genes of SARS-CoV-2 in one of the patients, and some fragments in other patients using SARS-CoV-2 RT-PCR (Fig. 3*A* and Dataset S3), suggesting that SARS-CoV-2 could exist in the gastrointestinal tract even when the primary infection-site lung had a very low viral load. We further examined the transcriptional response in the gastrointestinal tract by performing total RNA sequencing on the FFPE colon samples from these 9 fatal cases and 10 uninfected individuals as control. We found that although there is no obvious pathogenesis in the colon tissue based on histological examination, there is a dramatic change in the transcriptome in the colon tissues in the fatal cases when compared to healthy controls. With the caveat that the control samples represent histologically normal tissue obtained from patients with colon cancer, a total of 4,932 genes were identified as significantly differentially expressed genes, with 1,246 up-regulated and 3,686 down-regulated genes in the cases (Fig. 3*B*). Principal component analysis (PCA) of all samples from the two tissue sites revealed clear separation of the lung and colon in the controls, while the tissue-specific separation is not so clear in the cases (Fig. 3*C*). Consistent with the PCA, the pairwise Euclidean distances among the samples within the same tissue type is significantly lower than the pairwise Euclidean distances among the samples across the tissue types in the controls, while such comparison is no longer significant in COVID-19 cases (*SI Appendix*, Fig. S6).

We performed GO enrichment analysis on the colon tissues to compare the host response in these two sites (Fig. 3 *D* and *E*). There are very limited shared enriched pathways among these two sites, such as extracellular structure organization, “ossification,” and “maintenance of location among the up-regulated pathways.” The neutrophil signature is unique to the lung, suggesting that the massive increases of neutrophil infiltration into the lung are a primary characteristic of this site. Several enriched pathways in the up-regulated genes in the colon related to the response to the Transforming Growth Factor beta (TGF-β), suggesting that there is a local response to TGF-β in the colon. The only GO term enriched in the down-regulated genes and shared by the two tissue sites was “long-chain fatty acid catabolic process.” The top nine of the enriched GO terms in down-regulated genes involved in immune cell activation and immunity suggested the immune system is disrupted in other organs as well. Interestingly, the rest of the enriched GO terms in down-regulated genes are involved in metabolic processes, suggesting the metabolic function might be affected in COVID-19 patients. Formally, it is possible that some of these colonic transcriptional changes reflect the critically ill nature of these patients and are not specific to SARS-CoV-2 infection.

### Key Genes and Proteins in SARS-CoV-2 Viral Entry and Interactions in the Lung.

**Fig. 4.**
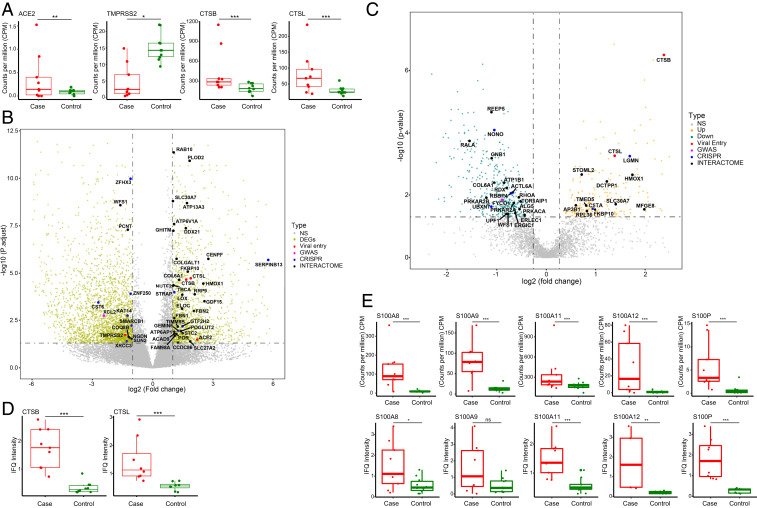
Key genes in SARS-CoV-2 pathogenesis are differentially expressed in mRNA and protein levels in fatal cases. (*A*) Gene expression levels of ACE2, TMPRSS2, and cathepsins B and L. * adjusted *P* < 0.05; ** adjusted *P* < 0.01; *** adjusted *P* < 0.001. Wald test in DESeq2, multiple test correction by BH. (*B* and *C*) Volcano plot (*P* value vs. fold change) comparing gene expression in cases vs. controls at mRNA level (*B*) and protein level (*C*). DEGs that were identified as important in host–SARS-CoV-2 interactions are highlighted based on the study in which they were identified and as described in the text. The horizontal dashed line represents an adjusted *P* value of 0.05, and the vertical dashed lines represent log2 FC of −1 and 1. Wald test in DESeq2, multiple test correction by BH. (*D*) Protein levels of cathepsins B and L. *** adjusted *P* < 0.001 Student’s *t* test. (*E*) Gene expression levels of mRNA and protein of S100A8, S100A9, S100A11, S100A12, and S100P. * adjusted *P* < 0.05; ** adjusted *P* < 0.01; *** adjusted *P* < 0.001; ns, not significant. Gene expression of mRNA was analyzed by Wald test in DESeq2. Protein levels were analyzed by Student’s *t* test.

To better integrate our findings with what is already known about SARS-CoV-2 pathogenesis in the lung, we examined expression of genes that have previously been implicated in viral entry. Angiotensin converting enzyme 2 (ACE2), the primary host receptor for SARS-CoV-2 entry (28), was increased by approximately fourfold in the lungs of patients with COVID-19 (Fig. 4*A*). TMPRSS2, a cellular serine protease that primes the spike protein of SARS-CoV-2 and has been shown to be important in facilitating viral entry into cells (28), is down-regulated in the lungs of fatal COVID-19 cases (Fig. 4*A*). Two other proteases, cathepsins B and L, which are able to substitute for TMPRSS2 (28), had increased expression (Fig. 4*A*), an observation that suggests these cathepsins might be the more relevant proteases for viral entry into the lung epithelium.

Additionally, in an effort to contextualize our transcriptome findings through the prism of functional genomics, we examined the expression level of genes that regulate SARS-CoV-2 pathogenesis as identified in a CRISPR screen (29), that interact with SARS-CoV-2 as assessed by protein–protein interactions (30), and that predispose to severe infection as characterized in a genome-wide association study (GWAS) (31) (Fig. 4*B* and Datasets S7–S9). Seven of the 104 genes identified in the CRISPR screen were up-regulated in our transcriptome data (ACE2, CTSB, CTSL, and SERPINB13 are proviral; STRAP, GTF2H2, and GEMIN6 are antiviral), and 6 genes were down-regulated (SMARCB1, ZFHX3, and CST6 are proviral; ZNF250, XRCC3, and KAT14 are antiviral). Of the 332 human proteins found to interact with SARS-CoV-2 proteins, 34 were differentially expressed in our dataset, with the overwhelming majority (29 genes) being up-regulated. None of the genes identified in the GWAS screen were differentially expressed in the lung tissue of fatal cases, but XCL2, a chemokine ligand of one of the hits (XCR1), was significantly down-regulated. This subset of genes that have been implicated in SARS-CoV-2 pathogenesis and have altered expression levels in the lung of severe cases may help guide future drug development for COVID-19.

To further validate the transcriptome observation and evaluate the host response at the protein level, we performed proteomics analysis using liquid chromatography with tandem mass spectrometry (LC-MS/MS) on these FFPE lung samples from cases and controls. In total, 4,689 proteins were detected by mass spectrometry, 4,635 of them are also expressed in the transcriptome data. We identified a total of 637 differentially represented proteins, with 235 up-regulated and 402 down-regulated proteins in the cases (Fig. 4*C* and Datasets S10–S12). The correlation coefficient between the significantly differential represented genes and proteins is 0.81 (*SI Appendix*, Fig. S7). Protease cathepsin B and L are also significantly highly represented in cases compared to the controls (∼5.2-fold and ∼2.6-fold increase, representatively; Fig. 4*D*). Several S100 proteins, such as S100 A8, A9, A11, A12, and P are more highly expressed and translated in the cases vs. controls (Fig. 4*E*). S100A8/A9 and S100A12, proinflammatory mediators, which are commonly found at inflammatory sites and in the serum of patients with inflammatory disease, contribute to modulate the local inflammatory response by stimulating leukocyte recruitment and induce cytokine secretion (32). These cytoplasmic proteins are mainly secreted by cells, such as neutrophils and macrophages, which is consistent with our observation of increased neutrophils and NETosis in the lung of fatal COVID-19 patients. Other studies have shown that serum levels of S100A8/A9 were significantly elevated in COVID-19 patients and strongly correlated with the severity of clinical manifestations (33–35). S100A8/9 is known to be expressed and secreted during infection-induced inflammation and is restricted by a negative feedback regulatory mechanism (36). Given the low viral load in these patients, the sustained elevation of S100A8/A9 level lends further support to the idea that an uncontrolled immune reaction contributes to the pathology. GO enrichment analysis of significantly up-regulated proteins identified four neutrophil-related GO terms as top enriched GO terms, as well as extracellular structure organization (*SI Appendix*, Fig. S8), consistent with the enriched GO pathways identified in the transcriptome data. Therefore, these highly expressed proteins such as S100 proteins, might represent potential therapeutic targets that facilitate regulation of the excessive inflammation response in COVID-19 patients with severe disease.

## Discussion

We have performed a detailed assessment of the host response to SARS-CoV-2 in human lung, a site of critical importance in SARS-CoV-2 pathogenesis, given the worldwide burden of morbidity and mortality stemming from COVID-19 pneumonia-related complications. Given that our data originate from a cohort of patients who died of COVID-19 in Wuhan, China, during the very beginning of the pandemic, these data will serve as an important resource that helps provide a baseline of the host response to the earliest SARS-CoV-2 strains against which future datasets can be compared. It is likely that mutations will continue to accumulate in the circulating SARS-CoV-2 strains, and the host response of future patient cohorts can subsequently be compared to this Wuhan dataset to better understand the evolution of host–virus interactions. Moreover, this Wuhan cohort represents the closest approximation to the “natural history” of COVID-19 that is likely to be studied, particularly since later patients—even in the early stages of the outbreak in the United States—were quickly being enrolled in a variety of clinical trials to assess different therapeutics.

The finding that these fatal cases had a low viral load in the setting of massive transcriptional changes is suggestive that patient deaths may be more related to an overexuberant and uncontrolled host response rather than unabated viral replication. This observation is consistent with what was seen in patients who died of SARS-CoV-1 and MERS-CoV infections and fits within the damage–response framework (37, 38), which holds that ongoing host damage may be the result of the host immune response rather than persistent microbial interactions. Moreover, our findings of a low viral burden at the time of fatal outcome may help explain why direct antivirals, such as remdesivir (39), are more effective in patients with less severe disease and dexamethasone, an immunosuppressant, is associated with reduced mortality in severely ill patients late in disease (40).

Ultimately, understanding the host response to SARS-CoV-2 in human lungs—the main site of infection leading to morbidity and mortality—may help inform future clinical trials for COVID-19 therapeutics. For example, our finding that expression of IL-6 is not increased in the lungs of these fatal cases highlights that anti-IL-6 therapies may not be an effective treatment for severe, late-stage COVID-19 pneumonia, which is consistent with preliminary randomized clinical trial findings (41, 42). The observation that neutrophils are transcriptionally enriched in the lungs with histologic evidence of NET formation may provide scientific justification for the 9 ongoing clinical trials testing the efficacy of dornase alfa. Moreover, the finding that expression of cathepsins B and L—but not TMPRSS2—is elevated in the lungs suggests that inhibitors of these proteases may help prevent viral entry (43). Continued study of the host response in concert with ongoing viral evolution will be critical to better understand these host–virus dynamics and provide a more complete understanding of COVID-19 pathogenesis.

## Materials and Methods

### Human Lung and Colon Samples.

Eight patients who died at Jinyintan Hospital (Wuhan, China) and one patient who died at Central Hospital of Wuhan as a result of COVID-19 underwent autopsies, during which samples were taken from representative areas of the lung and colon for routine histologic analysis. Clinical data regarding these deceased individuals were abstracted from the medical records. The control (uninfected) lung or colon samples (*n* = 10 for each control group) represent healthy tissue obtained from patients with lung cancer or colon cancer during biopsy and/or surgical resection performed as part of routine clinical care. These control patients were matched to the cases with respect to age, sex, and past medical history. Lung/colon tissues were fixed with 10% formalin for 3 d at room temperature, trimmed into appropriate size and shape, embedded in paraffin, and cut into 4-μm-thick sections for further histological and immunohistochemistry staining. The study protocol was approved by the Tongji Hospital affiliated to Tongji Medical College of Huazhong University of Science and Technology Institutional Review Board (approval TJ-IRB20200341).

### Immunofluorescence Staining for NETs.

The FFPE lung tissue sections were deparaffinized and rehydrated. Antigen retrieval was performed by heat and pressure with Tris-ethylenediaminetetraacetic acid (EDTA) buffer (pH 9.0) for 1.5 min. Endogenous peroxidase activity was blocked with 3% hydrogen peroxide for 15 min followed by blocking with 10% sheep serum for 15 min. The sample was incubated overnight at 4 °C with a 1:10,000 dilution of rabbit anti-human myeloperoxidase (clone EPR20257; Abcam), followed by a 45-min incubation at 37 °C with a 1:4,000 dilution of a polyclonal goat anti-rabbit IgG H&L (horseradish peroxidase [HRP]; Abcam) and a 10-min incubation at room temperature with a 1:400 dilution of a fluorescein isothiocyanate (FITC)-based tyramide signal amplification solution (Bios Biological; Wuhan, China). Slides were washed with tris-buffered saline (TBS) buffer, placed in Tris-EDTA buffer (pH 9.0), and heated for 10 min in a microwave for antigen retrieval. Samples were blocked with 10% donkey serum for 15 min, incubated overnight at 4 °C with a 1:200 dilution of a polyclonal rabbit anti-human neutrophil elastase (Abcam), and incubated at 37 °C for 45 min with a 1:400 dilution of a polyclonal Alexa Fluor 594-conjugated donkey anti-rabbit IgG (H&L) (Thermo Fisher). DAPI was used to stain DNA. Images were scanned using a Pannoramic MIDI (3DHISTECH; Budapest, Hungary).

### Library Preparation and Sequencing.

Total RNA was isolated from FFPE lung or colon tissue from cases and controls using the RNeasy FFPE kit (Qiagen) according to the manufacturer’s instructions with a minor modification. Thin tissue sections weighing ∼0.1 mg were used for each sample, and an additional genomic DNA removal step was performed by treating the sample with 2 units of TURBO DNA-free DNase (Invitrogen) for 30 min at 37 °C. The Trio RNA-Seq Library Preparation Kit (Nugen; Redwood City, CA) was used to generate sequencing libraries as per the manufacturer’s instructions, with 1 to 5 ng RNA used as input. The library size and quality were assessed using an Agilent Bioanalyzer 2100. Pooled libraries were sequenced on an Illumina NextSEq 550 using a 75-cycle kit with single-end read mode.

### SARS-CoV-2 Detection by RT-PCR.

Quantitative detection of SARS-CoV-2 in the RNA obtained from the FFPE samples was performed using the 2019-nCoV Real Time Triplex RT-PCR Kit (Liferiver Bio-Tech; Shanghai, China) per the manufacturer’s instructions. This kit employs primers specific for three different SARS-CoV-2 genes (the nucleocapsid [N], envelope [E], and RNA-dependent RNA polymerase [RdRp] genes) and requires that all three genes be detected with a cycle threshold ≤40 to conclude that SARS-CoV-2 is present. RT-PCR reactions were performed on 7500 Real-Time PCR Systems (Applied Biosystems).

### Bioinformatic Analysis.

Raw sequence reads were trimmed using Trim Galore (v0.6.4, https://github.com/FelixKrueger/TrimGalore) with parameters “-quality 20, -stringency 3, -length 20” to remove both poor quality calls and adapters. Trimmed reads were first aligned to the human reference genome hg19 using STAR 2.7.3a (44) with default parameters, and a quality control (QC) report was generated using RNA-SeQC 1.1.8 (45). A count table was generated via htseq_count using the parameters “-a 10, -m intersection-nonempty” using HTSEq 0.11.4 (46). Genes that were differentially expressed between cases and controls were characterized using DESeq2 (47) by identifying genes with |log2FC|>1 and a *P*-adjusted value <0.05.

Pathway analysis of the differentially expressed genes was performed using the R package clusterProfiler (48), with org.Hs.eg.db used for annotation. Enriched GO pathways were identified as GO terms that had a *P*-adjusted value <0.05 after Benjamini–Hochberg correction for multiple testing. One representative term was selected from redundant terms for a similarity >0.6 using the simplify function in clusterProfiler. The enriched GO terms were organized into a network with edges connecting overlapping gene sets with an overlapping threshold value of 0.2. The GO modules are the clusters of the GO terms with mutually overlapping gene sets, and they were named using the GO hierarchical structure in AmiGO2 (49).

Volcano plots were generated using the R package ggplot2. To assess expression of immune-related genes, we interrogated the genes that are described in Immport (https://www.immport.org/shared/genelists). To determine whether genes previously implicated as important in SARS-CoV-2 pathophysiology are differentially expressed in lung tissue of patients, we manually extracted gene lists from the literature (29–31) (Datasets S7–S12). All box and whisker plots were generated using the R package ggplot2. The box depicts the first to the third quartile, and the horizontal line indicates the median. Interquartile range (IQR) was calculated as the first quartile substracted from the third quartile. The whiskers extend to the most extreme dataset value that is within 1.5 times the IQR of the box. The data points outside the whiskers represent outliers.

Heatmaps were generated by using the R package pheatmap to plot the expression level of genes up-regulated in the Wuhan cases. For the heatmap of neutrophil-related genes, we included the genes in the GO terms in the GO network module “neutrophil-mediated immunity” as well as a published molecular signature of NETs (50). For the heatmap of extracellular structure organization-related genes, we included the genes in the GO terms in the GO network module named extracellular structure organization as well as fibrosis-related genes (downloaded from http://biokb.ncpsb.org/fibroatlas/index.php).

Principal component analysis was performed on the matrix containing the log transformation of the counts per million (cpm) reads for each gene across all cases and controls using the R function prcomp and plotted by ggbiplot, and the 95% confidence interval of groups was plotted as ellipses. Pairwise Euclidean distances among the samples within the same tissue type (intradistance) and across the tissue types (interdistance) were calculated in cases and controls separately, then multivariate analysis was performed using adonis function in R package Vegan (51) with 999 permutation.

### SARS-CoV-2 Mapping.

The reads that did not map to the human genome hg19 were filtered by read length <75 bp and aligned to the genome of SARS-CoV-2 isolate Wuhan-Hu-1 (National Center for Biotechnology Information [NCBI] NC_045512), using bowtie2 with default parameters (52). The reads that mapped to the SARS-CoV-2 genome were further filtered by alignment with mapping quality MAPQ <30 using samtools 1.9 (53), with the resulting reads representing the number of SARS-CoV-2 viral reads present in the sample. Read count per 100 bp was calculated using samtools 1.9.

### Protein Extraction and Digestion.

Protein extraction from FFPE tissues was performed according to previous methods (54, 55). Briefly, samples were dewaxed in xylene and pelleted by centrifugation. Pellets were resuspended in lysis buffer (1% sodium dodecyl sulfate [SDS], 200 mM Tris⋅HCl pH 8, 1% protease inhibitor mixture). After sonication for 5 min (3 s on, and 5 s off) at 30% amplitude, the tubes were heated at 100 °C for 30 min and subsequently centrifuged for 10 min at 20,000 × *g*. The supernatant containing the extracted proteins was recovered and quantified using a bicinchoninic acid (BCA) test. Proteins were reduced in 5 mM dithiothreitol (DTT) at 56 °C for 30 min and alkylated at room temperature in 11 mM iodoacetamide (IAA) for 15 min in the dark. Then the proteins were added to the filter membrane for filter-aided sample preparation. The membrane was washed three times with 8 M urea and then washed three times with 20 mM NH_4_HCO_3_ at 12,000 × *g*. A total of 100 μL of 20 mM NH_4_HCO_3_ containing trypsin (protein:trypsin = 50:1, m/m) was added to the membrane and incubated overnight at 37 °C. Peptides were harvested from the membrane by centrifuging for 10 min at 12,000 × *g* and then desalted using C18 precolumn and finally dried down using a vacuum centrifuge (Thermo). All samples were stored at −80 °C for further analysis.

### LC-MS/MS Analysis.

The tryptic peptides were dissolved in solvent A (0.1% formic acid in water), directly loaded onto a home-made reversed-phase analytical column (25-cm length, 75 μm internal diameter [i.d.]). Peptides were separated with a gradient from 4 to 6% solvent B (0.1% formic acid in acetonitrile) in 2 min, 6 to 24% over 68 min, 24 to 32% in 14 min, and climbing to 80% in 3 min, then holding at 80% for the last 3 min, all at a constant flow rate of 300 nL/min on a nanoElute high-performance liquid chromatography (UHPLC) system (Bruker Daltonics).

The peptides were subjected to capillary source followed by the timsTOF Pro (Bruker Daltonics) mass spectrometry. The electrospray voltage applied was 1.60 kV. Precursors and fragments were analyzed at the TOF detector, with a MS/MS scan range from 100 to 1700 *m/z*. The timsTOF Pro was operated in parallel accumulation serial fragmentation (PASEF) mode. Precursors with charge states 0 to 5 were selected for fragmentation, and 10 PASEF-MS/MS scans were acquired per cycle. The dynamic exclusion was set to 30 s.

### Proteomics Database Search.

The resulting MS/MS data were processed using Maxquant search engine (v.1.6.6.0). Tandem mass spectra were searched against the Uniprot human database (20,366 sequences, released at 2020/05) concatenated with reverse decoy database. Trypsin/P was specified as cleavage enzyme allowing up to two missing cleavages. The mass tolerance for precursor ions was set as 40 ppm in both first search and main search, and the mass tolerance for fragment ions was set as 0.04 Da. Carbamidomethyl on Cys was specified as fixed modification, and acetylation on protein N-terminal and oxidation on Met were specified as variable modifications. Peptide and protein false discovery rate (FDR) were adjusted to <1%.

## Supplementary Material

Supplementary File

Supplementary File

## Data Availability

The raw sequence data from the Wuhan cases and controls have been deposited to NCBI with BioProject accession no. PRJNA646224. All data processing was performed as described in *Materials and Methods* using publicly available software.
