# Supplementary material for: Transcriptional and proteomic insights into the host response in fatal COVID-19 cases
Source: Proc Natl Acad Sci U S A. 2020 Oct 20;117(45):28336–43. doi: 10.1073/pnas.2018030117 (PMC7668053; doi:10.1073/pnas.2018030117)
Supplement: Supplementary File [file pnas.2018030117.sapp.pdf]

PNAS
www.pnas.org

Supplementary Information for

Transcriptional and proteomic insights into the host response in fatal COVID-19 cases

Meng Wu[n,1], Yaobing Chen[a,1], Han Xia[b,c,1], Changli Wang[d,1], Chin Yee Tan[e,f,1] Xunhui Cai[g], Yufeng Liu[g], Fenghu Ji[g], Peng Xiong[g], Ran Liu[b], Yuanlin Guan[b], Yaqi Duan[a,d],Dong Kuang[a], Sanpeng Xu[a], Hanghang Cai[a], Qin Xia[h], Dehua Yang[i], Ming-Wei Wang[i], Isaac M. Chiu[n], Chao Cheng[j], Philip P. Ahern[k], Liang Liu[l,2], Guoping Wang[a,d,2], Neeraj K. Surana[e,f,m,2], Tian Xia[a,g,2], Dennis L. Kasper[n,2]

Corresponding authors: Liang Liu (Email: liuliang@mail.tjmu.du.cn)
Guoping Wang (Email: wanggp@hust.edu.cn)
Neeraj K. Surana (Email: neil.surana@duke.edu)
Tian Xia (Email: tianxia@hust.edu.cn)
Dennis L. Kasper (Email: dennis_kasper@hms.harvard.edu)

This PDF file includes:

Figures S1 to S8
Legends for Datasets S1 to S12

Other supplementary materials for this manuscript include the following:

Datasets S1 to S12

www.pnas.org/cgi/doi/10.1073/pnas.2018030117

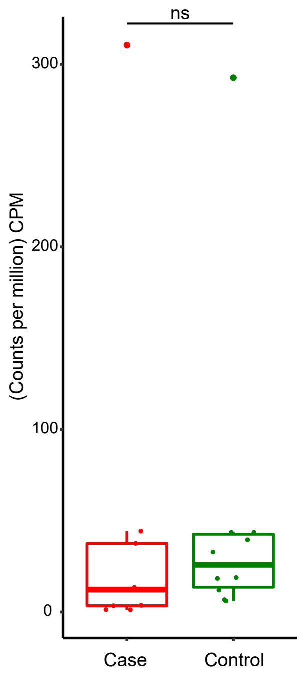

Figure S1. Gene Expression Level of IL-6 in Wuhan Cohort. *adjusted p <0.05; ns, non-significant. Wald test in DESeq2, multiple test correction by BH.

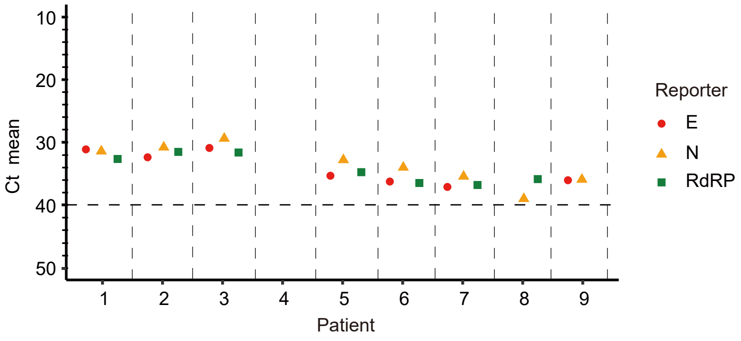

Figure S2. SARS-CoV-2 RT-PCR Results from the RNA Input for Transcriptional Profiling in the Lung. The three genes assayed include the nucleocapsid (N), envelope (E), and RNA-dependent RNA polymerase (RdRp) genes. The dotted line indicates the threshold for detecting a gene as present.

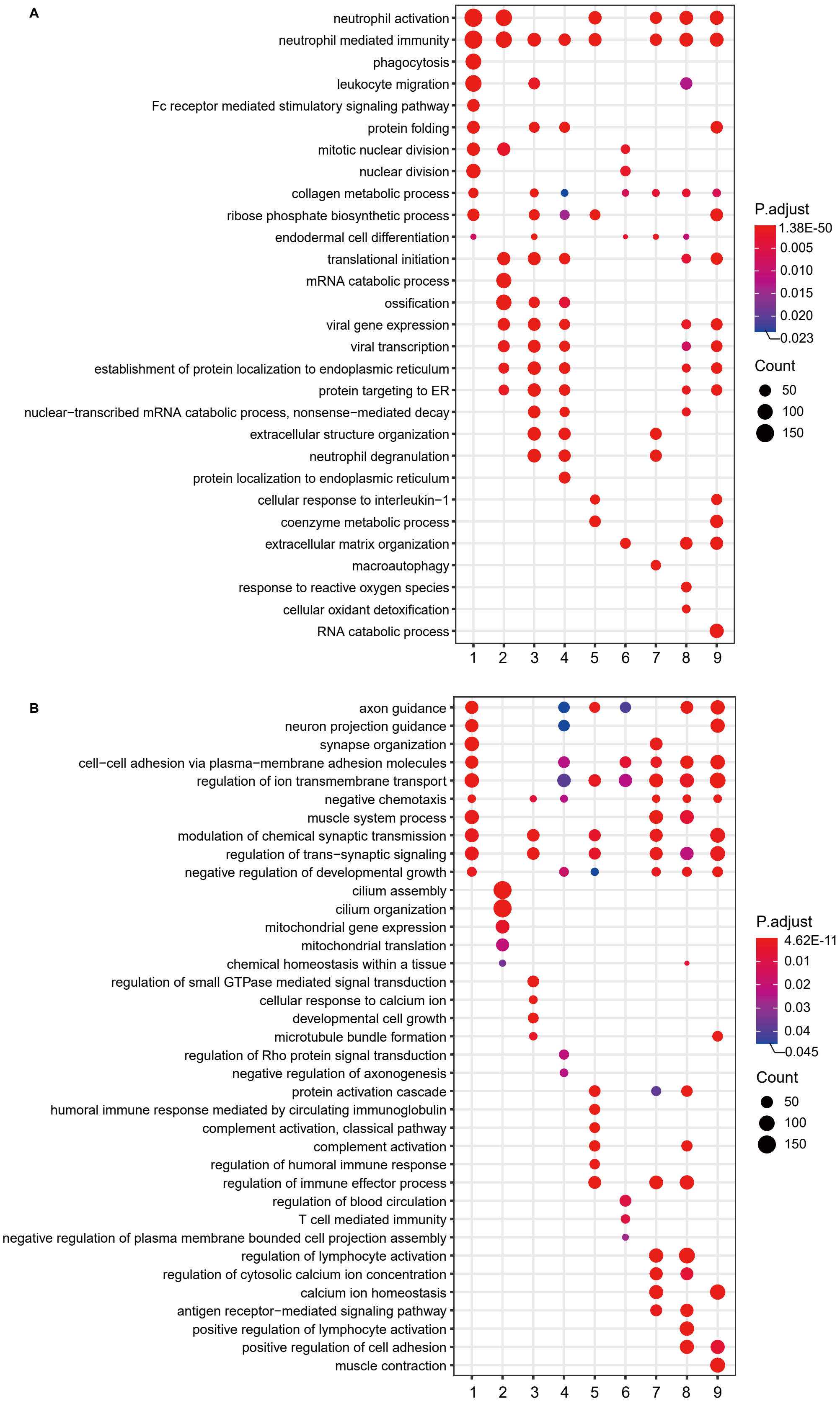

Figure S3. GO Term Analysis for Each Patient in the Wuhan Cohort. Dotplot visualization of enriched GO terms of up- *(A)* and downregulated *(B)* DEGs in cases. The color of the dots represents the p-value adjusted by Benjamini-Hochberg for each enriched GO term identified by Fisher's exact test using enrichGO function in R package clusterProfiler, and the size of the dot represents the number of genes enriched in the total gene set.

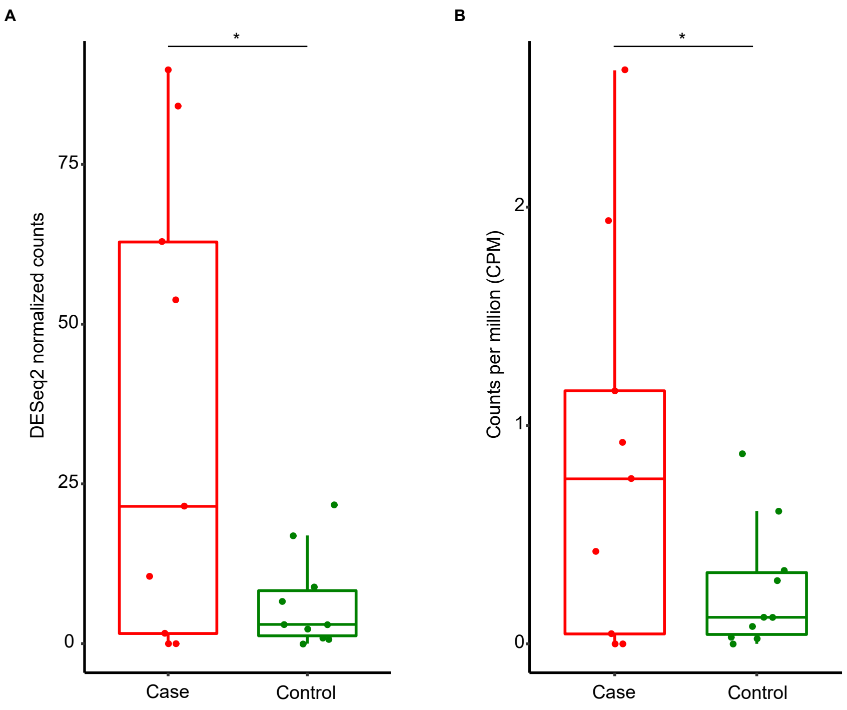

Figure S4. Gene Expression Level of Platelet Factor 4 (PF4). *(A)* Read counts have been normalized by DESeq2. *(B)* Read counts are depicted as counts per million. * adjusted p <0.05. Wald test, multiple test correction by BH.

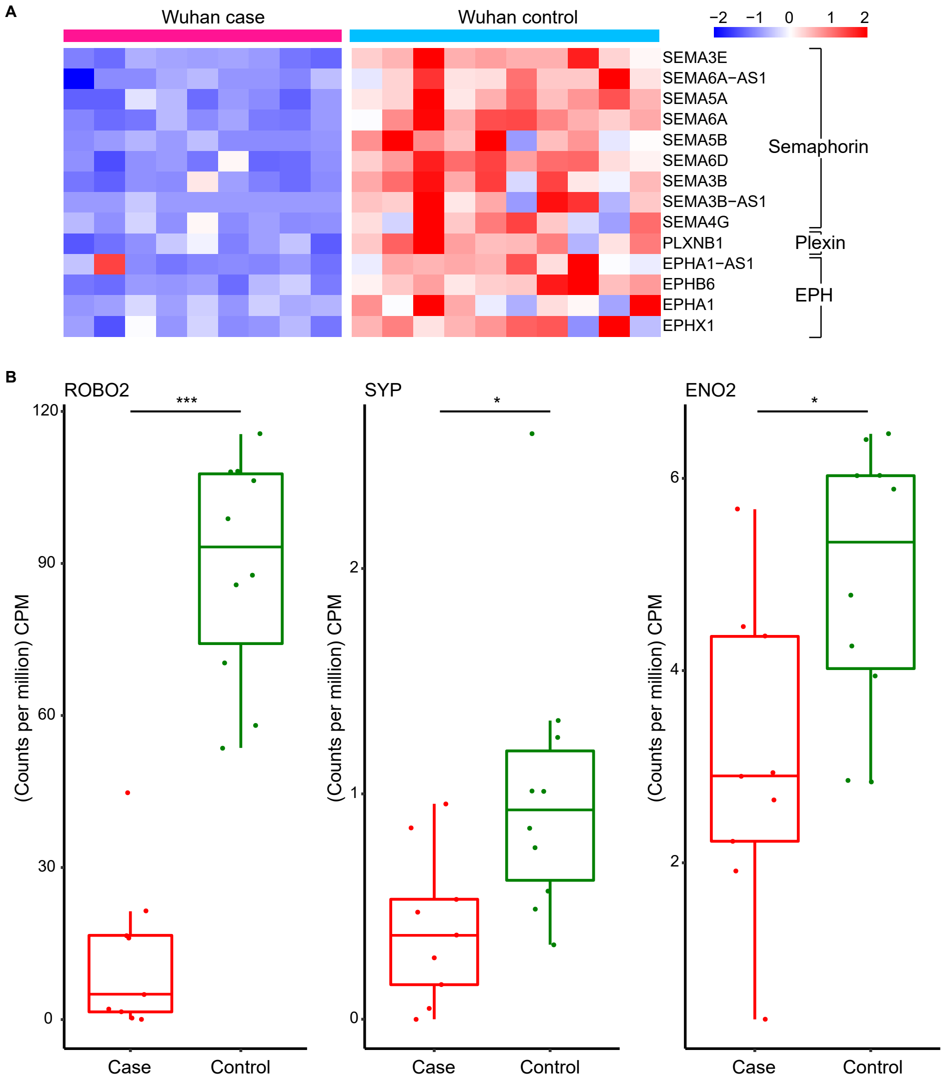

Figure S5. Comparison of Select Neurobiology-Related Genes. *(A)* Heatmap indicating the expression levels of downregulated DEGs in semaphorins, plexin, and ephrin receptors. *(B)* Gene expression levels of ROBO2, SYP, and ENO2, all of which are markers for pulmonary neuroendocrine cells. *adjusted p <0.05; ***adjusted p <0.001. Wald test in DESeq2, multiple test correction by BH.

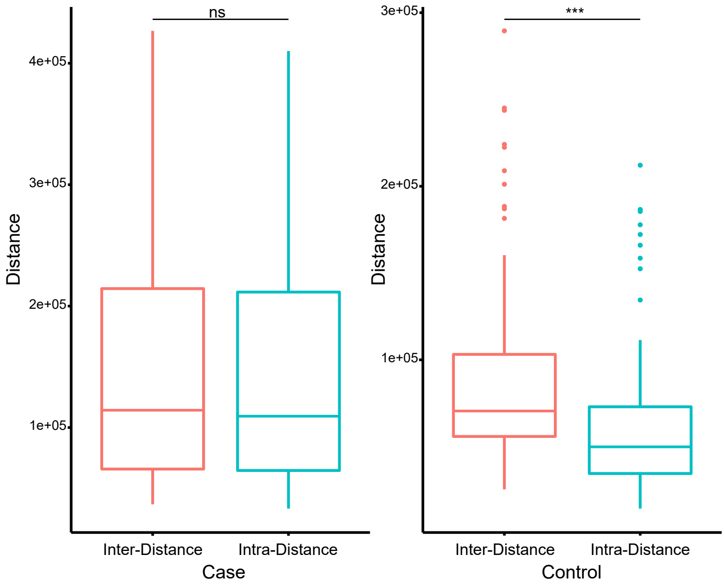

Figure S6. Comparison of Transcriptome Distances Within and Across Tissues in Cases and Controls. Inter-distance: pairwise distances among samples across lung and colon. Intra-distance: pairwise distances among samples within lung or colon. ***adjusted p <0.001; ns non-significant. Permutation test with pseudo-F ratios.

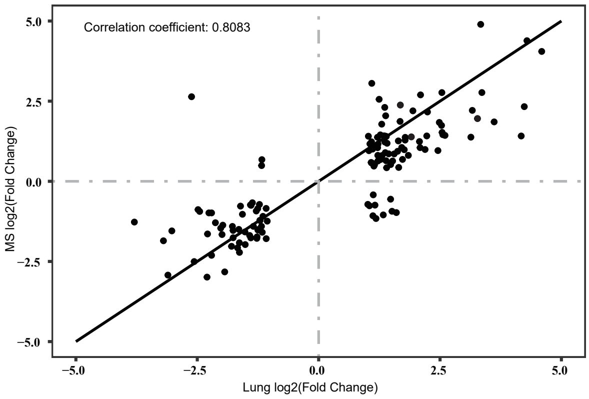

Figure S7. Correlation of Differentially Expressed Genes at mRNA and Protein Levels Using Log Fold Changes.

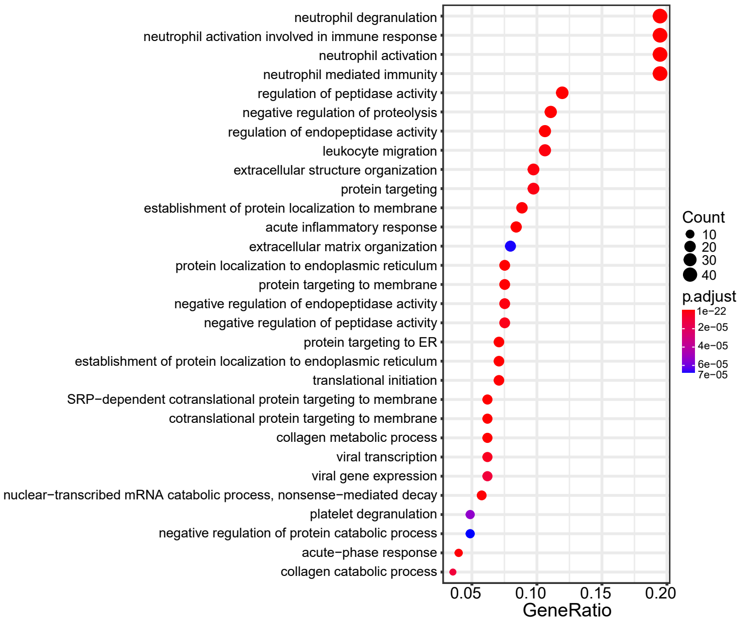

Figure S8. GO Term Analysis for Up-Regulated Proteins in the Lung of COVID19 Patients Dotplot visualization of the top 30 enriched GO terms of upregulated proteins. Fisher's exact test using enrichGO function in R package clusterProfiler.

Dataset S1. Detailed Clinical Information for Patients in the Wuhan Cohort

Dataset S2. Differential Expressed Genes Involved in Immune-Related Pathways

Dataset S3. Viral Reads Present in the Sequencing Data

Dataset S4. Quality Control Report for RNAseq Analysis of Samples from Wuhan Cohort

Dataset S5. Upregulated DEGs Involved in Neutrophil Activation, Neutrophil Mediated Immunity, and NETs

Dataset S6. Upregulated DEGs Involved in Fibrosis and Extracellular Structure Organization

Dataset S7. DESeq2 Results for Genes Identified in A CRISPR Screen

Dataset S8. DESeq2 Results for Genes Identified Via Interactome Studies

Dataset S9. DESeq2 Results for Genes Identified in A GWAS, Along with Their Associated Chemokine Ligands

Dataset S10. Mass Spectrometry Results for Genes Identified in A CRISPR Screen

Dataset S11. Mass Spectrometry Results for Genes Identified Via Interactome Studies

Dataset S12. Mass Spectrometry Results for Genes Identified in A GWAS, Along with Their Associated Chemokine Ligands
